# A Modern Treatment for Pyorrhea

**Published:** 1919

**Authors:** 


**A Modern Treatment for Pyorrhea.** By Lieut. J. W. SANBORN. *American Dentist*, October 1, 1918.

In the treatment of pyorrhea, patients should undergo a complete physical examination, including urinalysis, blood count, blood pressure, etc. The whole mouth should be radiographed to show any chronic alveolar abscesses, blind abscesses absorbed, or eroded roots, the amount of attachment of the tooth, also any pieces of filling pushed between the teeth, and other sources of irritation. All of these abnormal conditions must be remedied. Both physical examinations and radiograph should be repeated in six months. The operator must use his own judgment in regard to extraction of any teeth. If a tooth can be rotated, or if under pressure it returns as if pressed against a cushion, it should be extracted.

All causes of irritation, such as ill-fitting crowns, faulty fillings, etc., should be removed. In real pyorrhea there is always formation under the gum margin. This must all be removed by careful surgery with the proper instruments, which should extend to all portions of exposed roots. Great care is necessary not to scratch or gouge the surface. By use of tape and wood points with silex, these surfaces must be brought to as high a polish as the enamel itself; all exposed surfaces should be so smooth that the point of an explorer will glide over them as over glass. Under this treatment the inflammation and swelling of the gums reduces. Medical treatment hastens the process. Alcresto ipecac tablets, Dunlop paste, and Packer's, or Buckley's astringent may be used; suction cups are also recommended. Strong mouth washes should not be used habitually. All teeth should be treated, those in best condition first until fermentation is reduced.

